# Unplanned reoperation after resection of retroperitoneal sarcoma: experience based on a high-volume sarcoma center

**DOI:** 10.1186/s12957-022-02633-y

**Published:** 2022-05-18

**Authors:** Aobo Zhuang, Mingkun Zhao, Yuan Fang, Lijie Ma, Weiqi Lu, Yuhong Zhou, Yong Zhang, Hanxing Tong

**Affiliations:** 1 Department of General Surgery, Zhongshan Hospital, Fudan University, Shanghai, China; 2 Department of General Surgery, South Hospital of the Zhongshan Hospital/Shanghai Public Health Clinical Center, Fudan University, Shanghai, China; 3 Department of Medical Oncology, Zhongshan Hospital, Fudan University, Shanghai, China

**Keywords:** Retroperitoneal sarcoma, Surgery, Unplanned reoperation, Quality indicator

## Abstract

**Background:**

Most retroperitoneal sarcoma (RPS) operations require combined multi-organ resection, and the proportion of unplanned reoperation is high. However, there are no relevant studies on reoperation for RPS.

**Methods:**

Patients who underwent at least once unplanned reoperation at Shanghai Public Health Clinical Center, Fudan University, China, from August 2009 to December 2021 were retrospectively analyzed. The baseline characteristics, primary surgery, and reoperation information, postoperative complications, and survival were analyzed.

**Results:**

A total of 51 patients were included. Among them, 21 (41.2%) were male and 30 (58.8%) were female. The median age was 51 (interquartile range [IQR], 49-63) years. Most (88.3%) had a history of abdominal surgery. Dedifferentiated liposarcoma, well-differentiated liposarcoma, leiomyosarcoma, and others accounted for 50.9%, 21.6%, 15.7%, and 11.8%, respectively. The conditions of the primary operation were as follows: 35 (68.6%) patients achieved complete surgical resection, 48 patients had combined organ resection, and a median of 3 (IQR, 2–4) organs was removed, of which 5 (9.9%) were combined with pancreaticoduodenectomy. The median operative time was 330 (IQR, 245–440) min, and the median estimated blood loss was 1500 (IQR, 500–2600) ml. The median postoperative hospital stay was 42 (IQR, 23–82) days. For reoperation, the most common reasons were bleeding (31.3%), complications related to intestinal anastomosis (27.4%), and intestinal perforation (19.9%). The mortality rate after reoperation was 39.2% (20/51). Twelve (23.5%) patients underwent reoperation at least twice.

**Conclusions:**

Unplanned reoperation among retroperitoneal sarcoma correlates with established measures of surgical quality.

## Background

Soft tissue sarcoma is a rare group of cancers consisting of more than 50 histologic subtypes of mesenchymal origin. With approximately 15,000 new diagnoses per year [1], soft tissue sarcoma remains an exceedingly rare but a manageable and treatable disease. In the absence of effective adjuvant therapy, surgery is the best option [2]. However, resection of retroperitoneal soft tissue sarcoma (RPS) represents a particular challenge. Given its proximity to vital structures and the generally large size of these tumors [3, 4], complete resection often requires combined multiple organ removal [5, 6], which makes resection of RPS a particular challenge.

Existing studies have pointed out that the incidence of severe postoperative adverse events after RPS is about 15%, and the incidence of unplanned reoperation is about 10% [7, 8]. In comparison, the proportion of reoperation in conventional general surgery is about 3.5% [9], and even in pancreaticoduodenectomy, the proportion of reoperation is only about 6% [10].

Reoperation not only causes more trauma for patients, but also increases their economic and psychological burden and requires much more medical resources because of the prolonged hospital stay. Therefore, reoperation has been included in the reference index of the Joint Commission on the Accreditation of Healthcare Organizations for medical injury and medical quality assessment [11]. Despite interest in using reoperation as a quality indicator, its impact on patient outcomes and costs has not been carefully described in RPS surgical practice. Furthermore, little is known about the cause of reoperation occurrence, and how often they reflect technical failures related to the procedures themselves. Information about its causes is critical for quality improvement efforts aimed at reducing the incidence of reoperation.

Therefore, the purpose of this study was to take advantage of our high-volume sarcoma center to analyze the causes and outcomes of postoperative reoperation in patients with RPS and provide a reference for the surgical management of RPS patients.

## Methods

All RPS patients surgically treated at the South Hospital of the Zhongshan Hospital/Shanghai Public Health Clinical Center, Fudan University, Shanghai, China, from August 2009 to December 2021 were reviewed. Inclusion criteria were as follows: (1) underwent unplanned reoperation, (2) retroperitoneally located primary tumor, (3) sarcoma confirmed by pathology, (4) over 18 years old, and (5) complete follow-up data. Patients suffering from Ewing sarcoma, alveolar/embryonic rhabdomyosarcoma, desmoid tumors, gynecologic sarcoma, and gastrointestinal stromal tumors were excluded. This study was approved by the Ethics Committee of the South Hospital of Zhongshan Hospital/Shanghai Public Health Clinical Center, and it was conducted following the principles of the Declaration of Helsinki.

Tumor burden was the sum of the largest diameters of all tumors described in the surgical record. Surgical resection was classified as complete resection (R0 or R1) and incomplete resection (R2). Postoperative morbidity was graded based on the Clavien-Dindo classification [12]. Clavien-Dindo classification greater than or equal to III is defined as major postoperative complications. Histological subtypes were assigned as follows: well-differentiated liposarcoma, dedifferentiated liposarcoma, leiomyosarcoma, and others (including malignant peripheral nerve sheath tumor, solitary fibroma, undifferentiated pleomorphic sarcoma, synovial sarcoma, and fibrosarcoma). Tumor grades were assigned in accordance with the Federal National Cancer Center (FNCLCC) grading system. The physical status of patients before anesthesia was assessed according to the American Society of Anesthesiologists Physical Status (ASA score).

All patients were operated on by the same sarcoma-specific surgical team. And all patients were followed up postoperatively until hospital discharge or for 30 days. Unplanned reoperation (our primary outcome measure) was defined as any secondary surgery required to develop complications directly or indirectly from index surgery. Therefore, planned reoperations at the time of index surgery or subsequent surgery not related to surgical complications were not accounted for. The postoperative follow-up included clinical and imaging examinations (contrast-enhanced CT or contrast-enhanced magnetic resonance imaging from the chest to the pelvis). Follow-up was required every 3 months for the first 2 years postoperatively, every 6 months thereafter, as well as once a year after 5 years. Furthermore, disease progression was assigned as imaging-diagnosed new lesions or marked enlargement of the original lesions.

### Statistical analyses

Progression-free survival (PFS) and overall survival (OS) rates were determined using the Kaplan-Meier method. Postoperative deaths were excluded from the survival analysis. Quantitative data were reported as median (interquartile range). Qualitative data were reported as number of patients (percentage of patients). All analyses were performed using the SPSS Statistics (IBM SPSS Statistics for Windows, Version 23.0. Armonk, NY, United States: IBM Corp.).

## Results

### Patients and tumor characteristics

Fifty-one patients met the enrollment criteria. There were 21 (41.2%) males and 30 (58.8%) females with a median age of 51 (interquartile range [IQR], 49–63) years. Twenty-two (43.2%) patients had a preoperative ASA score of more than 2, and most patients (88.3%) had previous abdominal/pelvic surgery. Forty-two (82.3%) patients were recurrence disease, and 3 (5.9%) patients had metastatic disease. The median tumor burden was 20 (IQR, 16–25) cm. Most patients (76.4%) were symptomatic at presentation, and about half (41.2%) had multifocal disease. There were well-differentiated liposarcoma, dedifferentiated liposarcoma, leiomyosarcoma, and others in 26 (50.9%), 11 (21.6%), 8 (15.7%), and 6 (11.8%) patients, respectively. Twenty-two (43.2%) patients received adjuvant therapy before surgery (Table 1).

**Table 1:** Patients and tumor characteristics in the 51 patients with unplanned reoperation

Characteristics	*N*=51	% of total
Gender		
Male	21	41.2
Female	30	58.8
Age, years median (IQR)	51	49–63
ASA score		
1–2	29	56.8
>2	22	43.2
Current smoker		
Yes	3	5.9
No	48	94.1
Current drinker		
Yes	2	3.9
No	49	96.1
History of abdominal/pelvic surgery		
Yes	45	88.3
No	6	11.7
Tumor resection times		
Metastatic disease		
Yes	3	5.9
No	48	94.1
Times of sarcoma resection		
First	9	17.7
Second	16	31.4
More than twice	26	50.9
Symptoms at visit		
Yes	39	76.4
No	12	23.6
Tumor burden, cm median (IQR)	20	16–25
Multifocality		
Yes	21	41.2
No	30	58.8
Histologic subtypes		
Dedifferentiated	26	50.9
Well-differentiated	11	21.6
LMS	8	15.7
Others	6	11.8
FNCLCC		
Grade 1	11	21.6
Grade 2	26	51.0
Grade 3	13	25.5
Unknown	1	1.9
Preoperative radiation		
Yes	1	1.9
No	50	98.1
Radiation		
Yes	8	15.7
No	43	84.3
Preoperative chemotherapy		
Yes	11	21.6
No	40	78.4
Chemotherapy		
Yse	20	39.3
No	31	60.7

### Primary surgical characteristics

All patients (100%) underwent open surgery, and 68.6% had a complete resection. For surgical procedures, 3 (5.9%) patients had mass resection only, 10 (19.7%) patients had diaphragmatic reconstruction, 11 (21.6%) patients had major vascular surgery (including the abdominal aorta, inferior vena cava, and iliac vessels), and 5 (9.9%) patients underwent pancreaticoduodenectomy. The median number of resected organs was 3 (IQR, 2–4), and the most common organ resected was colon (76.4%), followed by the jejunal and ileal (50.9%) and kidney (23.5%). The median operative time was 330 (IQR, 245–440) min. The median estimated blood loss was 1500 (IQR, 500–2600) mL. 76.4% of patients required intraoperative packed RBC transfusion, with a median transfusion of 4 (IQR, 4–8) units. Most patients (92.1%) were transferred to the ICU after surgery. The median postoperative hospital stay of all patients was 42 (IQR, 23–82) days (Table 2).

**Table 2:** Primary surgical characteristics of the 51 patients with unplanned reoperation

Characteristics	*N* = 211	% of total
Operation		
Laparoscopic surgery	0	0
Open surgery	51	100.0
Complete resection		
Yes	35	68.6
No	16	31.3
Mass excision only		
Yes	3	5.9
No	48	94.1
Diaphragmatic excision and reconstruction		
Yes	10	19.7
No	41	80.3
Abdominal wall excision and reconstruction		
Yes	7	13.8
No	44	86.2
Vascular surgery		
Yes	11	21.6
No	40	78.4
Gynecologic surgery		
Yes	7	13.8
No	44	86.2
Pancreaticoduodenectomy		
Yes	5	9.9
No	46	90.1
Number of combined resections median (IQR)	3	2–4
Resected organs		
Colon	39	76.4
Jejunal and ileal	26	50.9
Kidney	12	23.5
Pancreas	11	21.5
Diaphragm	10	19.6
Duodenum	10	19.6
Spleen	6	11.8
Adrenal gland	5	9.8
Operative time, hours median (IQR)	330	245–440
Estimated blood loss, ml median (IQR)	1500	500–2600
Packed RBC transfusion		
Yes	39	76.4
No	12	23.6
Packed RBC transfusion, unit median (IQR)	4	4–8
ICU stay		
Yes	47	92.1
No	4	7.9
Postoperative hospital stay, days median (IQR)	42	23–82

### Characteristics of reoperation

The most common reasons for reoperation were postoperative bleeding (31.3%), bowel anastomotic-related complication (27.4%), and bowel fistula (19.9%), followed by incision-related complications (7.8%), bile leak/fistula (5.8%), and others (7.8%). The median time interval from primary surgery to reoperation was 7 (IQR, 4–14) days. Specifically, there were 6 cases within 24 h, and of them, 3 cases were due to abdominal hemorrhage, 2 cases were due to bowel fistula, and one case was due to biliary leakage. Most patients underwent reoperation 1–7 days after primary surgery (23 cases, 45.0%), of which 8 were due to bowel anastomotic leakage, 6 were due to hemorrhage, 5 were due to bowel fistula, 2 were due to biliary leakage, and 2 were due to incision-related complications. Seventeen (33.4%) patients underwent reoperation 7–30 days after surgery, and of them, 6 were due to bowel anastomotic leakage, 6 were due to abdominal hemorrhage, 1 was due to bowel fistula, 1 was due to incision-related complications, and 3 were due to other reasons (1 of pancreatic fistula, 1 of adnexal infection, and 1 of compartment syndrome). There were 5 patients who received reoperation more than 30 days after primary surgery, and of them, 2 were due to bowel fistula, 1 was due to hemorrhage, 1 was due to incision-related complications, and 1 was due to urinary fistula. Major postoperative complications occurred in 30 (59.0%) patients, and of them, 12 (23.5%) underwent a second reoperation. Twenty patients (39.2%) died postoperatively (Table 3).

**Table 3:** Characteristics of the 51 patients with unplanned reoperation

Characteristics	*N*=51	% of total
Reasons for the unplanned reoperation		
Postoperative bleeding	16	31.3
Bowel anastomotic-related complications	14	27.4
Bowel fistula	10	19.9
Incision-related complications	4	7.8
Bile leak/fistula	3	5.8
Others	4	7.8
The interval between primary operation and reoperation		
24 hours	6	11.7
1–7 days	23	45.0
7–30 days	17	33.4
More than 30 days	5	9.9
Clavien–Dindo classification		
NA	8	15.6
1–2	13	25.4
3–5	30	59.0
Second reoperation		
Yes	12	23.5
No	39	76.4
Postoperative death		
Yes	20	39.2
No	31	60.8

All reoperations due to postoperative bleeding were intra-abdominal hemorrhage, of which 68.7% were due to rupture from direct arterial or venous trauma, and the rest (31.3%) were due to erosion of a vascular structure. Three reoperations were performed within 24 h, and 13 beyond. There were 3 (18.8%) cases treated by interventional surgery ahead of surgery, and the bleeding was not alleviated after the intervention. 25.0% of patients experienced a second reoperation. The postoperative mortality rate was 50.0% (8/16). Of the 14 patients with bowel anastomotic-related complications who underwent reoperation, 8 (50.0%) had 1 anastomosis, 7 (43.7%) had 2 anastomoses, and 1 (6.3%) had 3 anastomoses. In terms of the location of anastomotic leakage, 6 (37.5%) cases were intestinal anastomotic leakage, 3 (18.8%) cases were ileocolonic anastomotic leakage, 2 (12.5%) cases were colonic anastomotic leakage, and 5 (31.2%) cases were stump fistula. About 50% of the anastomotic leakage occurred 1–7 days after surgery, while the other half occurred 7–30 days after surgery. 35.7% of patients experienced a second reoperation, and 7 (50.0%) patients died postoperatively. There were 10 patients who underwent reoperation because of bowel fistula, and all of them had small intestinal perforation. Two of the perforation sites were repaired with sutures in the primary operation. The vast majority (90.0%) of reoperation occurred within 30 days of the surgery. One (10.0%) patient underwent a second reoperation, and 2 (20.0%) patients died postoperatively.

### Survival analysis

**Fig. 1:**
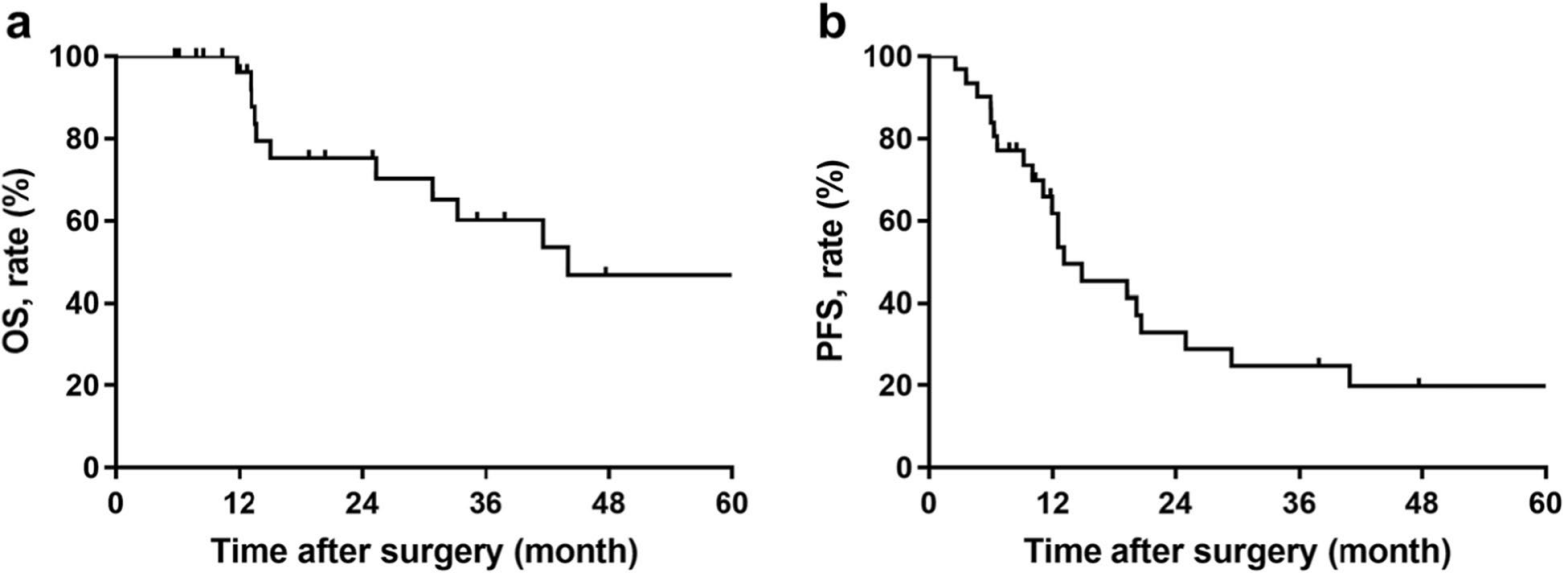
**a** Overall survival and **b** progression-free survival in patients with unplanned reoperation

Excluding 20 patients who died postoperatively, the other 31 patients were routinely followed up. At a median follow-up of 37.9 (95% CI 23.1–52.8) months, nine patients were still alive without disease progression, and twelve patients died from the disease. The 5-year OS rate and 5-year PFS rate were 46.8% (95% CI 23.9–69.7%) and 19.8% (95% CI 3.7–35.9%), respectively (Fig. 1).

## Discussion

RPS is still a disease that relies on surgical treatment. Previous studies have reported in detail the incidence of perioperative complications and related risk factors for RPS. According to a report from the Transatlantic RPS Working Group (TARPSWG), the incidence of reoperation after surgery is about 10% [7, 8], which is even higher than pancreaticoduodenectomy. However, there are currently no relevant reports focusing solely on the reoperation of RPS. This study reviewed the 10-year experience of 51 patients in a high-volume sarcoma center and found that the most common reasons for reoperation were postoperative bleeding (31.3%), followed by bowel anastomotic-related complications (27.4%), and bowel fistula (19.9%). Reoperation occurred in 90% of the patients within 30 days of the primary surgery, and 23.5% of them experienced a second reoperation. The postoperative mortality of patients undergoing reoperation with RPS was as high as 40%. For patients who did not experience postoperative death, the 5-year OS rate and 5-year PFS rate were 46.8% and 19.8%, respectively, which were worse than primary RPS [7] but comparable to locally recurrent RPS [8].

In 2018, a cohort study consisting of more than 1000 RPS patients from the TARPSWG indicated that reoperation was required in 10.5% of patients. Although no risk factors for reoperation were reported, the study indicated that age, transfusion requirements, and resected organ score were risk factors for major postoperative complications [7]. Also from the TARPSWG, a cohort study based on 681 locally recurrent RPS patients reported that 12% of patients experienced reoperation, and the risk factor associated with major postoperative complications was transfusion requirement [8]. Of the 249 patients with primary RPS who underwent active surgical treatment at two major European referral centers, 12% required reoperation. Reoperation was performed in 50.0% of patients due to anastomotic leakage, 20.0% due to postoperative bleeding, 16.7% due to retroperitoneal abscess, and 13.3% due to incision-related complications [13]. In 2010, the National Surgical Quality Improvement Program (ACS-NSQIP) analysis reported a reoperation rate of 4.5% in 156 patients, but only 37% of their cohort underwent combined organ resection and indications for reoperation were not abstracted in ACS-NSQIP [14]. Another report utilizing the ACS-NSQIP, which included 564 patients, noted that only 3.9% of RPS patients experienced reoperation, which was in contrast to previous studies. Similarly, only 41.3% of patients in this study underwent combined organ resection, and no reason for reoperation was reported either [15]. In 2015, a retrospective analysis of 362 patients who underwent surgery at the Royal Marsden Hospital showed that 80.7% underwent combined excision and 7.4% underwent reoperation. However, the study did not address the reasons for reoperation [16]. Recently, Meredith et al. and Li et al. reported RPS combined with pancreatectomy and the reoperation rates were 14.0% and 22.2%, respectively. However, the sample sizes of the two studies were only 50 and 27 [17, 18].

From the above, we can see that the proportion of reoperation in RPS patients is significantly higher than that in general surgery. For pancreatoduodenectomy, the reoperation rate is about 6% [19], but when combined with RPS resection, it becomes three times more likely to be reoperation. In addition, there are differences in the reasons for reoperation compared with abdominal surgery. A study based on 3044 patients reported that the three most common causes for reoperation were incision-related complications (19.6%), anastomotic leakage (15.0%), and infection (15.0%) [9]. In our cohort, the three most common causes were bleeding (31.3%), anastomotic-related complications (27.4%), and bowel fistula (19.9%). There are three main reasons for the above differences. Firstly, RPS is always diagnosed at an advanced stage and is thus very large, and the median size of the total tumor in this study was 20 cm, which resulted in a larger surgical scope and a larger number of combined organ resections. In this study, the median number of organ resections was 3, in TAPSWG’s study the median number of organ resections in 1007 patients was 2 [20]. More organ removal means longer operation time and more postoperative complications [17]. Secondly, their malnourished status. 82.3% of the patients in this cohort had recurrent disease, and multiple surgeries also aggravated the malnutrition state. It has been reported that more than 50% of patients with retroperitoneal liposarcoma have malnutrition, and malnutrition status is associated with increased perioperative morbidity [21]. Thirdly, due to the limited efficacy of chemotherapy and radiation, resection of a local recurrent tumor may also be considered for well-selected patients. Therefore, a large proportion of patients with retroperitoneal tumors may have a history of multiple surgeries. Nearly 90% of the patients in the reoperation cohort had a history of abdominal surgery. Patients with multiple operations are often accompanied with severe intra-abdominal adhesions, and the release of intra-abdominal adhesions is often accompanied with unexpected intestinal damage. As reported by Richard et al., 10.5% of patients undergoing adhesiolysis inadvertently incurred bowel defect, and adhesiolysis was associated with an increase of sepsis incidence, intra-abdominal complications, and wound infection. Morality after adhesiolysis in the presence of a bowel defect was 8% while it was only 1.6% after uncomplicated adhesiolysis [22].

Postoperative bleeding is the most common cause of reoperation. Early arterial hemorrhage has been reported to have a better prognosis than late hemorrhage, and immediate repeated laparotomy is considered the mainstay of treatment. However, late postoperative emergency laparotomy is associated with higher morbidity and mortality [23]. In this study, the proportion of patients with bleeding more than 24 h after surgery accounted for 82.3%, and the mortality rate was as high as 53.8% (7/13) for late postoperative bleeding. With the advancement of interventional radiology, angiography and transcatheter arterial embolization have been widely used as an alternative to reoperation in the diagnosis and treatment of arterial hemorrhage after abdominal surgery [24], in the clinical practice of our center, the first choice for postoperative bleeding is interventional therapy not surgery.

Among the 51 patients, 12 underwent at least unplanned surgery twice, and the postoperative mortality was 39.2% (20/51). We believe that the high mortality rate of unplanned reoperation is mainly due to the following two reasons: First, compared with tumors in the abdominal cavity, RPS often involves important organs and requires combined multi-organ resection which may lead to a higher perioperative mortality. A study on the perioperative safety of RPS by Marko et al. reported a 90-day mortality rate as high as 10.4% [25]. As a high-volume sarcoma center, the situations of patients we deal with can be more complex. 43.2% of the patients had an ASA score greater than 2, and 88.3% of the patients had a history of abdominal and pelvic surgery, and the median number of organ resections was 3 (Tables 1 and 2). These factors increased the risk of postoperative mortality. On the other hand, reoperation-associated increases in postoperative mortality in patients with abdominal surgery have long been reported [26–28]. In general surgery, reoperation was associated with a seven-fold increase at the rate of postoperative mortality compared with patients who did not undergo reoperation [9]. Specifically, reoperation mortality after pancreatectomy was 25% [26], and reoperation mortality after liver resection was 17% [27]. A cohort study consisting of 1558 cases receiving postoperative unplanned reoperation for colorectal cancer also pointed out that the 30-day mortality rate of patients who received one unplanned reoperation after colorectal cancer surgery was 10.5%, and patients who received more than one reoperation was 28.2% [28]. In this study, 20 patients died in the postoperative period, but 11 died more than 30 days after surgery, so the 30-day mortality was 17.6% (9/51), which was comparable to the above study.

Over the past decade, a radical surgical approach involving en bloc resection of the sarcoma with adherent organs or structures has been advocated at many centers with the aim of minimizing marginality and increasing the local control [29, 30]. Although studies have confirmed that extended resection is not associated with increased postoperative complications, the high mortality rate of reoperation of RPS still needs to be paid enough attention to. In order to reduce reoperation, we give several advice based on our experience: (1) Establish a multidisciplinary mechanism and make sure multidisciplinary consultation is applied in all decision-making processes. The chief surgeon is a professional sarcoma surgeon with rich experience. According to the needs of the operation, our MDT team also includes vascular surgeons, thoracic surgeons, cardiac surgeons, urologists, orthopedists, obstetricians, and plastic surgeons; (2) For multiple recurrent diseases, the decision for re-resection should be made with caution and based on the patient’s status, recurrence-free interval, and tumor biology. And our surgical strategy is to minimize unnecessary surgical trauma for recurrence disease; (3) The 10 patients with postoperative intestinal perforation in this cohort all had a history of abdominal/pelvic surgery and were accompanied with severe intra-abdominal adhesions. Therefore, for patients with severe intra-abdominal adhesions, and without short bowel syndrome, we prefer to perform a resection of the adherent bowel instead of release; and (4) In order to reduce unnecessary trauma and reoperation, retroperitoneoscopic biopsy may be a good option for patients who require open biopsy [31]. We stress again that the treatment of RPS requires going to a high-volume sarcoma center. A larger center with experienced practitioners and the necessary resources can provide safe and high-value healthcare to patients undergoing high-risk surgical procedures. Growing experience in the management of postoperative complications in evolving specialist units and providing the necessary resources for interdisciplinary care may further decrease operative mortality after RPS resection.

With the maturity of surgical techniques, the mortality rate after RPS has been reduced to about 2% [7, 8, 32]. However, surgery remains extremely complex, with various surgical techniques and complications, and reoperation is relatively non-discretionary compared to other potentially broad measures of quality such as wound infection (patients often return to the operating room only when a genuine need exists) and relatively discrete events. Again, they are easy to track through medical history, so reoperation rates may help monitor medical treatment in sarcoma centers and serve as a quality control indicator for RPS surgery.

This study has the following shortcomings. First, the retrospective nature and a long time span of the study could cause potential selection bias, and the sample size is only 51 cases, so the conclusions of the study need to be treated with caution. Second, as mentioned earlier, malnutrition status may be associated with reoperation, but due to lack of information, nutritional indicators were not included in this study. Third, this study only explored the reasons for unplanned reoperation and patient outcomes, and did not investigate the risk factors of reoperation. It is necessary to include patients who did not undergo reoperation in the future to enrich the study. Finally, because of the complexity of unplanned reoperations and potentially more complex patient admissions at our center as a high-volume sarcoma center, the applicability of the conclusions drawn from this cohort may be limited.

## Conclusions

In conclusion, the most common causes of unplanned reoperation for RPS are postoperative bleeding, bowel anastomotic-related complications, and bowel fistula, with a median hospital stay of 42 days and a postoperative mortality rate of 39.2%. Unplanned reoperation can be used as a quality control tool for RPS surgery.

## Data Availability

The datasets used and analyzed during the current study are available from the corresponding author on reasonable request.

## Abbreviations

RPS: Retroperitoneal sarcoma
IQR: Interquartile range
FNCLCC: The Federal National Cancer Center
PFS: Progression-free survival
OS: Overall survival
TARPSWG: The Transatlantic RPS Working Group
ACS-NSQIP: The National Surgical Quality Improvement Program
